# No Tillage During the Summer Fallow Enhanced Soil Functional Quality by Regulating Soil Structure and Organic Carbon Sequestration

**DOI:** 10.3390/plants15050791

**Published:** 2026-03-04

**Authors:** Qingshan Yang, Yuanyuan Yong, Qian Hu, Changxin Han, Zhenping Yang, Zhiqiang Gao, Jianfu Xue

**Affiliations:** College of Agriculture, Shanxi Agricultural University, Key Laboratory of Sustainable Dryland Agriculture of Shanxi Province, Taigu 030801, China; b20221011@stu.sxau.edu.cn (Q.Y.); yongyuanyuan@sxau.edu.cn (Y.Y.); 20232186@stu.sxau.edu.cn (Q.H.); 20230100307@stu.sxau.edu.cn (C.H.); yangzhenping340@163.com (Z.Y.)

**Keywords:** soil physical properties, soil aggregate, organic carbon sequestration, yield, Z-score

## Abstract

To address the issue of inefficient soil water utilization in dryland wheat fields, caused by a mismatch between summer fallow precipitation and crop growth periods, implementing fallow-period tillage was crucial for conserving water and enhancing yield. However, there was a lack of comprehensive evaluations of the impact of different tillage practices on soil functional quality based on multidimensional indicators, and the relationship between yield and soil functional quality remained unclear. This study established three treatments during the summer fallow period: no tillage (FNT), subsoiling tillage (FST) and plowing tillage (FPT). We determined the soil water-stable aggregates particle size distribution and stability, aggregate organic carbon (AOC) content, soil organic carbon (SOC) content and storage (SOCs), as well as winter wheat yield. Using the Z-score method, we integrated the soil’s physical and chemical indicators to perform a comprehensive evaluation of different tillage practices. The results showed that FNT significantly enhanced soil aggregate stability in the 0–30 cm soil depths compared to FST and FPT (*p* < 0.05), which was primarily attributed to a substantial increase in the content of >2 mm aggregates. Meanwhile, FNT resulted in significantly higher SOCs within the 0–50 cm profile, with increases of 8.1% and 5.8% compared to FST and FPT (*p* < 0.05), respectively. This was primarily due to elevated SOC content and higher AOC contents within the 2–0.25 mm and >2 mm aggregates in the topsoil layer. In contrast, FST significantly increased grain yield compared to FNT and FPT, by 16.7% and 15.0% (*p* < 0.05), respectively, which was associated with higher ear number and ear grains. A comprehensive evaluation using the Z-score method revealed that FNT achieved the highest soil functional quality score across the five layers. Therefore, no tillage during the summer fallow can enhance soil functional quality, primarily due to its positive impact on soil structure and carbon sequestration, but may not immediately increase crop yield.

## 1. Introduction

Soil aggregates, as the fundamental structural units of soil, are vital for maintaining a healthy pore structure, water and nutrient retention, and erosion resistance [1]. In agricultural systems, tillage practices significantly influence the formation, turnover, and stability of these aggregates, thereby regulating soil organic carbon (SOC) sequestration and cycling [2]. However, conventional tillage such as plowing has been shown to exacerbate drought stress, deplete soil resources, increase yield variability, and potentially lead to soil degradation over time [3]. Therefore, in modern sustainable agricultural development, the effectiveness of farmland management measures must be evaluated not only in terms of winter wheat (*Triticum aestivum* L.) yield but also in terms of how they enhance soil health, ecosystem services, and food production capacity.

Tillage practices and the addition of external organic matter, such as crop residues, were crucial in regulating aggregate dynamics [4,5]. Compared to conventional tillage, no tillage (NT) combined with straw incorporation can substantially raise the proportion of soil water-stable large aggregates (>0.25 mm), thereby enhancing aggregate stability [6,7]. Although subsoiling tillage improved deep soil structure, its physical protection of aggregates may be weaker than that under NT [8,9]. Conversely, plowing tended to disrupt macro-aggregates and accelerate organic matter decomposition, thereby reducing SOC content [10,11]. Mean weight diameter (MWD), geometric mean diameter (GMD) and fractal dimension (D) were commonly used to characterize soil aggregate stability [12,13]. Most studies indicated that conservation tillage practices (such as NT or reduced tillage) can increase MWD [14,15]. Nevertheless, this phenomenon manifested considerable regional heterogeneity; for instance, studies conducted in the Mediterranean region had reported contradictory trends [16], suggesting that the impact of tillage on aggregate stability was not universally consistent.

Soil aggregates organic carbon (AOC) content often responded more sensitively to management practices than bulk SOC content [17]. Numerous studies have confirmed that NT increased the OC content of large aggregates [18]. In particular, Kumar et al. [19] reported that NT increased OC content in silt and clay fractions by 45% and 74% at depths of 0–20 cm and 20–40 cm, respectively. Moreover, subsoiling tillage has been shown to promote carbon accumulation in deep soil aggregates [20,21]. Nonetheless, other studies suggest that in regions such as Australia and the Mediterranean, conservation tillage may improve aggregate structure without significantly increasing SOC storage (SOCs) or AOC content [16,22]. Additionally, research indicated that deep plowing promoted the SOC storage by increasing deep storage space [23]. Generally, intense soil disturbance is detrimental to soil aggregate formation and carbon sequestration [24]. Despite these insights, a systematic understanding of how various tillage practices during the summer fallow influence AOC content distribution, SOC content and storage in dryland wheat fields remained unclear.

The influence of tillage on crop yield was mediated through its effects on soil moisture and physical structure [25]. It was recognized that NT can enhance water use efficiency and yield by improving soil water retention and fostering root growth [26]. However, it was also noted that NT may reduce soil temperature, potentially delaying seedling emergence and negatively impacting yield [27]. In some arid regions, NT has even been associated with yield reductions of 12–18% [28]. Conversely, subsoiling tillage effectively broke through the plow pan, improved soil permeability and deep-water storage, and offered significant advantages in stabilizing and enhancing yields during drought years [29,30]. Consequently, the uncertain impact of tillage on yield was equally constrained by specific climate and soil conditions.

Existing research has primarily focused on analyzing the individual effects of tillage practices on either soil carbon sequestration or crop yield enhancement, but has lacked a comprehensive analysis that integrates the “soil structure–organic carbon sequestration–crop yield” framework. To address this gap, this study implemented different tillage practices during the summer fallow in dryland wheat fields, with the following objectives: (1) to determine how tillage practices affected soil aggregate size distribution, stability, and AOC content; and (2) to quantitatively evaluate tillage performance using a composite soil functional quality based on Z-scored integration of soil physical properties, aggregate characteristics, and carbon storage, and explore the relationship between yield and soil functional quality. To this end, we hypothesized that: (1) FNT could improve aggregate size distribution and stability, and increase AOC content within macro-aggregates; and (2) owing to its combined benefits for soil structure and carbon sequestration, FNT will achieve the highest comprehensive soil functional quality score in the multi-indicator evaluation and relatively high yield.

## 2. Results

### 2.1. Soil Physical Properties

**Figure 1:**
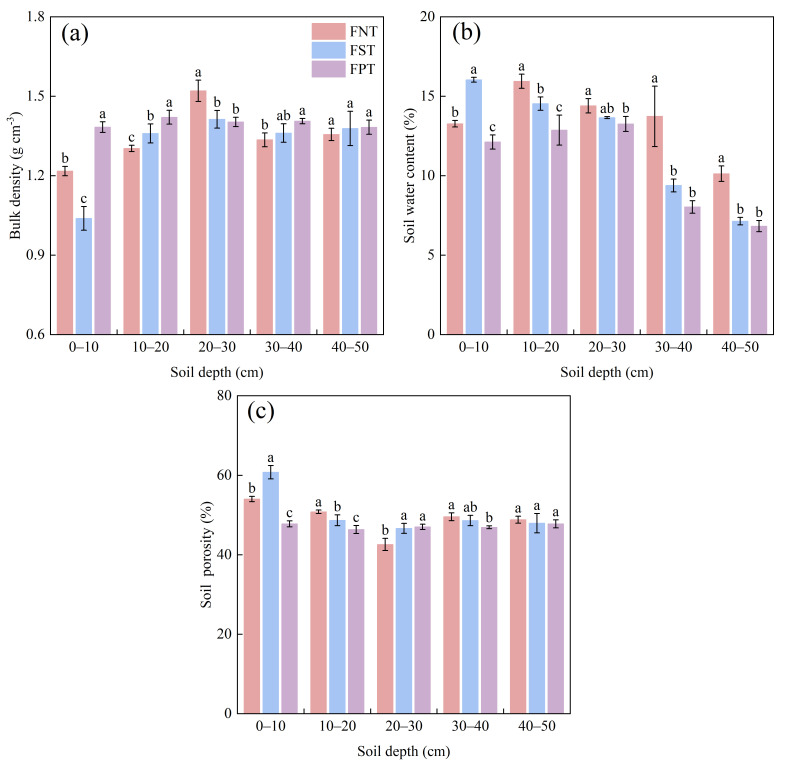
Impact of different tillage during the summer fallow on soil physical properties: (**a**) Bulk density (g cm^−3^); (**b**) Soil water content (%); (**c**) Soil porosity (%) across five soil depths during the 2025 winter wheat harvest season. Values show the mean (n = 3) ± SD (standard deviation). Significant differences among three tillage practices are indicated by lowercase letters on the same soil layer (*p* < 0.05, Duncan’s test). FNT = no tillage during the summer fallow; FST = subsoiling tillage during the summer fallow; FPT = plowing tillage during the summer fallow.

It was observed that BD in the 0–20 cm soil depths under FNT and FST were substantially lower than under FPT, reaching 8.3–12.0% and 4.3–24.9%, respectively. Meanwhile, the BD was highest under the FNT at a soil depth of 20–30 cm. BD under FNT was significantly lower than under FPT (5.0%) at a soil depth of 30–40 cm (Figure 1a). SWC and SP were highest in the FST across the 0–10 cm soil depth. FNT significantly increased SWC in the 10–20 cm and 30–50 cm soil depths by 9.7–46.4% and 23.9–71.0%, compared to FST and FPT, respectively (Figure 1b). At soil depths of 10–20 cm and 30–40 cm, the SP sequence was FNT > FST > FPT, with SP under FNT significantly higher than under FPT (5.7–9.6%). However, SP was lowest in the 20–30 cm soil depth under FNT (Figure 1c).

### 2.2. Particle Size Distribution and Stability of Water-Stable Aggregate

**Figure 2:**
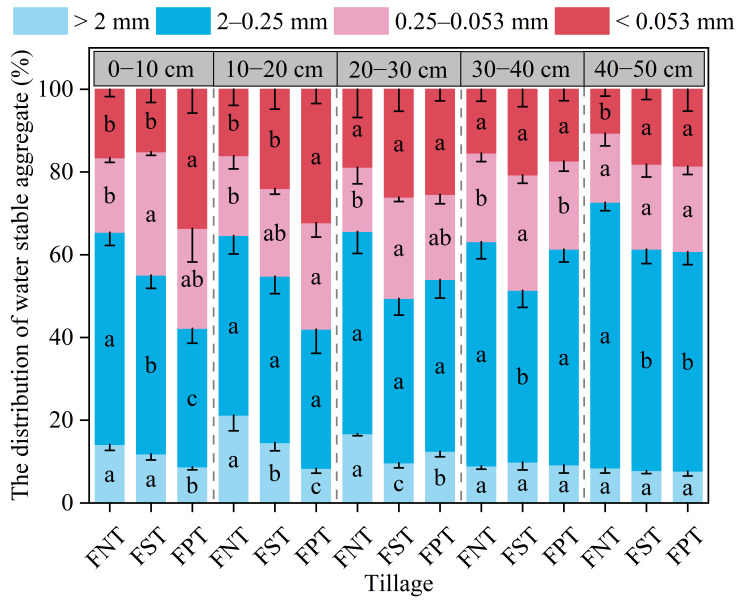
Impact of different tillage on particle size distribution and stability of water-stable aggregate during the summer fallow during the 2025 winter wheat harvest season. Values show the mean (n = 3) ± SD. Significant differences among three tillage practices are indicated by lowercase letters on the same soil layer (*p* < 0.05, Duncan’s test).

We found that compared to FST and FPT, FNT demonstrated a notably higher content of >2 mm aggregates in the 0–30 cm soil depths, with increases of 19.3–72.7% and 33.9–153.8%, respectively (Figure 2). The content of 2–0.25 mm aggregates in the 0–10 cm and 40–50 cm soil layers was significantly higher under FNT than under FST (18.7–20.0%) and FPT (21.0–53.5%). The content of 2–0.25 mm aggregates in the 30–40 cm soil layer was lowest under FST. Compared to FNT, both FST and FPT increased the content of 0.25–0.053 mm aggregates in the 0–30 cm soil depths by 9.9–65.5% and 32.4–34.5%, respectively, with a significant difference not observed between FST and FPT. In contrast, the content of aggregates < 0.053 mm was highest under FPT at soil depths of 0–20 cm, where FPT increased it by 34.5–122.3% relative to FNT and FST.

**Figure 3:**
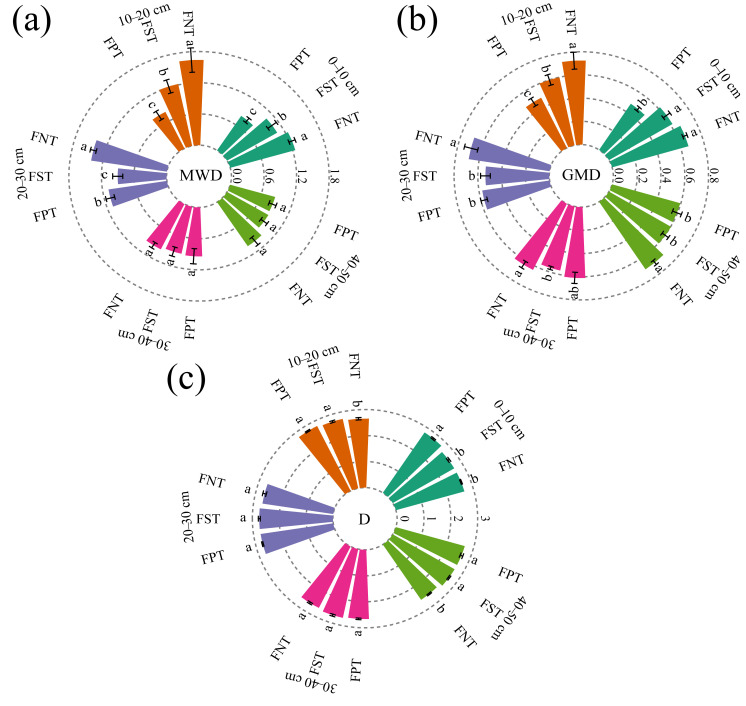
(**a**) Mean weight diameter (MWD); (**b**) geometric mean diameter (GMD); (**c**) fractal dimension (D) under different tillage practices and five soil layers during the 2025 winter wheat harvest season. Values show the mean (n = 3) ± SD. Significant differences among three tillage practices are indicated by lowercase letters on the same soil layer (*p* < 0.05, Duncan’s test). Five colors represent five soil layers.

Our findings indicated that FNT significantly improved soil aggregate stability compared to FST and FPT. Specifically, FNT increased the MWD value across the 0–30 cm soil depths by 16.7–56.8% and 29.8–108.6% (Figure 3a), and increased the GMD value in the 10–50 cm soil depths by 12.7–28.1% and 4.2–49.9% (Figure 3b), respectively. In contrast, the D value was lowest under FNT in the 10–20 cm and 40–50 cm soil depths, with FNT reducing the D value by 2.8–4.1% and 4.1–4.9% compared to FST and FPT, respectively. However, the D value was highest under FPT treatment at the 0–10 cm soil depth (Figure 3c).

### 2.3. AOC Content Within Water-Stable Aggregates, SOC Content and Storage

**Figure 4:**
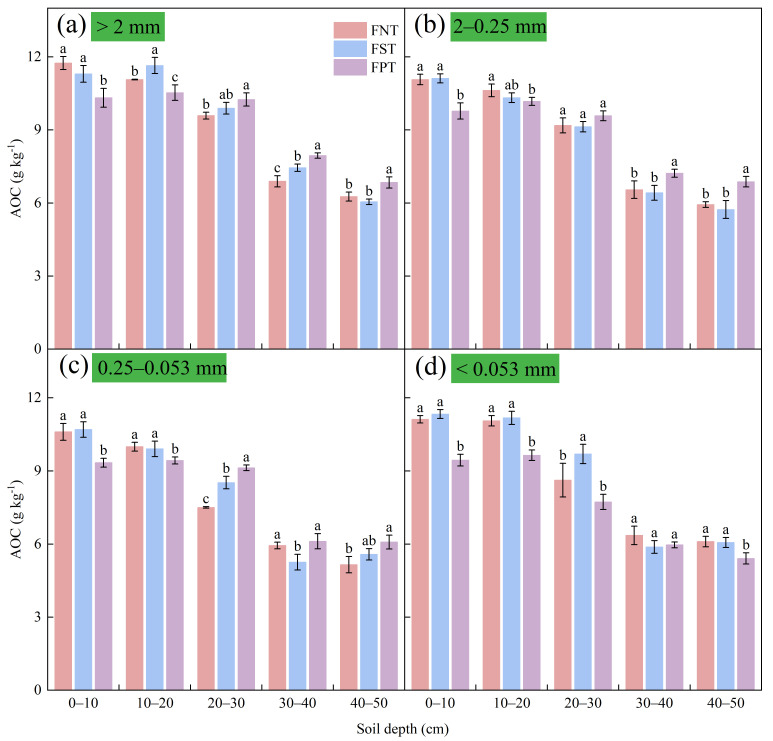
Impact of different tillage during the summer fallow on AOC (g kg^−1^) in four water-stable aggregate sizes: (**a**) >2 mm; (**b**) 2–0.25 mm; (**c**) 0.25–0.053 mm; (**d**) <0.053 mm during the 2025 winter wheat harvest season. Values show the mean (n = 3) ± SD. Significant differences among three tillage practices are indicated by lowercase letters on the same soil layer (*p* < 0.05, Duncan’s test).

Compared to FPT, FNT and FST significantly increased AOC content in aggregates > 2 mm in the 0–20 cm soil depths by 5.1–13.8% and 9.5–10.6%, respectively. Conversely, in the 30–50 cm soil depths, FPT showed higher AOC content in aggregates > 2 mm than FNT (9.3–15.3%) and FST (6.7–13.1%) (Figure 4a). A substantial rise in AOC content within 2–0.25 mm aggregates was observed under FNT and FST in the 0–10 cm soil layer, reaching 13.2–13.7%, compared to FPT. However, at soil depths of 30–50 cm, the AOC content of this aggregate size was significantly higher under FPT than under FNT (10.3–15.9%) and FST (12.5–19.9%) (Figure 4b). FNT and FST exhibited statistically significant increases in AOC content within 0.25–0.053 mm aggregates in the 0–20 cm soil layers, reaching 5.9–13.5% and 5.0–14.5%, respectively, compared to FPT. However, the highest AOC content within this particle size aggregate was found under FPT at soil depths of 20–50 cm (Figure 4c). The AOC content in aggregates < 0.053 mm was significantly higher under FNT and FST than under FPT in the 0–20 cm and 40–50 cm layers, with values ranging from 12.8% to 17.7% for FNT and 12.1% to 19.9% for FST (Figure 4d).

**Figure 5:**
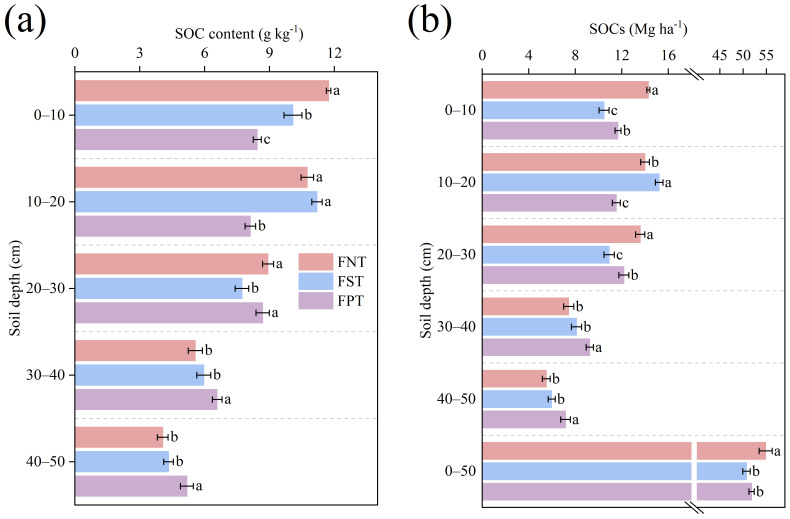
Impact of different tillage during the summer fallow on (**a**) SOC content (g kg^−1^); and (**b**) SOC storage (SOCs) (Mg ka^−1^) in the 0–50 cm soil depths during the 2025 winter wheat harvest season. Values show the mean (n = 3) ± SD. Significant differences among three tillage practices are indicated by lowercase letters on the same soil layer (*p* < 0.05, Duncan’s test).

Figure 5a results showed that compared to FST and FPT, SOC content in the 0–10 cm layer was substantially higher under FNT by 16.4% and 39.1%, respectively. Conversely, in the 30–50 cm layers, SOC content was significantly increased under FPT, exceeding FNT by 18.2–27.5% and FST by 10.4–19.6%. Similarly, SOCs in the 0–50 cm soil layer under FNT were significantly higher than those under FST (8.1%) and FPT (5.8%). However, FPT significantly enhanced SOCs by 24.4–30.0% and 14.0–20.0% at soil depths of 30–50 cm, compared to FNT and FST, respectively (Figure 5b).

### 2.4. Wheat Yield and Its Component Factors

It was evident that the FST achieved the highest yield, which was significantly higher than that of the FNT (16.7%) and FPT (15.0%) (Table 1). Compared to FNT and FPT, the FST treatment significantly increased both the number of ears and grain ears by 4.0–8.9% and 9.9–10.0%, respectively. Nevertheless, the FPT treatment exhibited the highest thousand-grain weight.

### 2.5. Correlation Analysis of Soil Physical Properties, Aggregate Characteristics, OC Indicators, and Yield

**Figure 6:**
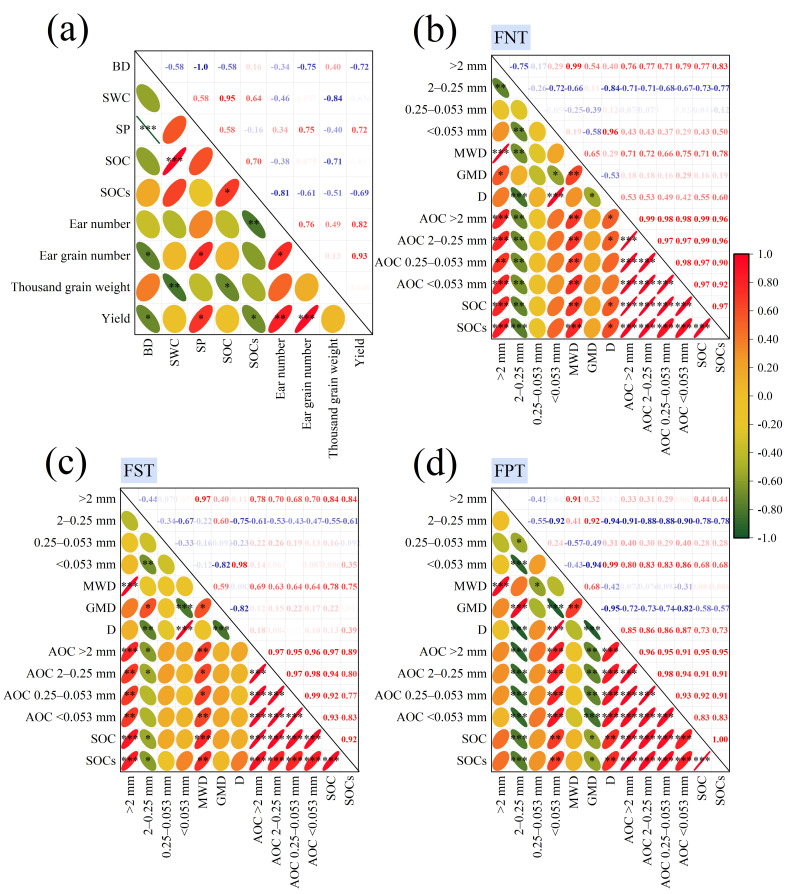
Correlation heatmap showed (**a**) relationship among soil physical properties, carbon indicators, yield, and its components; (**b**) relationship among aggregate characteristics, OC in aggregates, and carbon indicators under FNT; (**c**) relationship among aggregate characteristics, OC in aggregates, and carbon indicators under FST; (**d**) relationship among aggregate characteristics, OC in aggregates, and carbon indicators under FPT. Red and green (or blue) colors mean positive and negative correlations. *, **, and *** denote significant differences at *p* < 0.05, *p* < 0.01, and *p* < 0.001, respectively.

**Figure 7:**
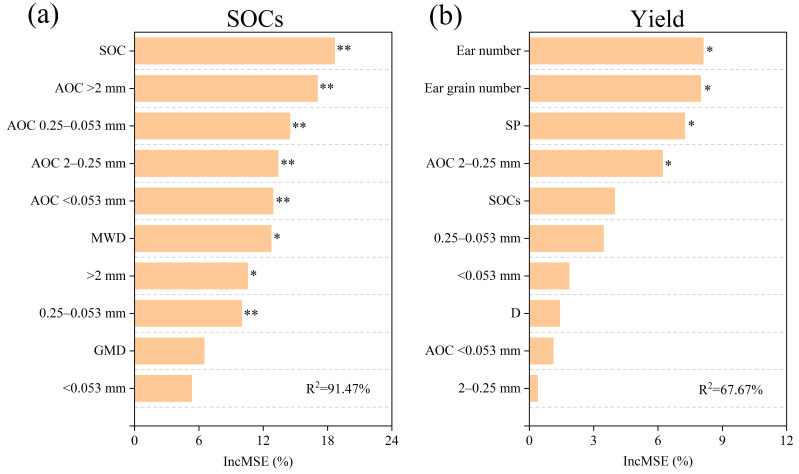
The importance of different explanatory variables for the (**a**) SOCs and (**b**) yield. * and ** mean significant differences at *p* < 0.05 and *p* < 0.01, respectively. R^2^ represents the degree of variance explained.

The correlation heatmap revealed that yield and ear grain number were positively correlated with SP, and ear number, whereas negatively correlated with BD. SOCs showed positive correlations with SOC content, but negative correlations with ear number and yield. SOC content correlated positively with SWC, whereas it correlated negatively with thousand-grain weight (Figure 6a). Under FNT and FST, SOC content, and SOCs were positively correlated with the content of >2 mm aggregates, MWD, and AOC content within all aggregate fraction sizes, while negatively correlated with the content of 2–0.25 mm aggregates (Figure 6b,c). Conversely, under FPT, SOC content, and SOCs were positively correlated with content of <0.053 mm aggregates, D, and AOC content within all aggregate fraction sizes, whereas negatively correlated with the content of 2–0.25 mm aggregates and GMD (Figure 6d). However, the order of Pearson’s correlation coefficients between SOC content and AOC content in four particle size aggregates was FNT > FST > FPT (Figure 6). Random forest modeling identified SOC content and ear number as the most critical factors influencing wheat yield and SOC storage, respectively (Figure 7).

### 2.6. Comprehensive Assessment of Soil Functional Quality

**Figure 8:**
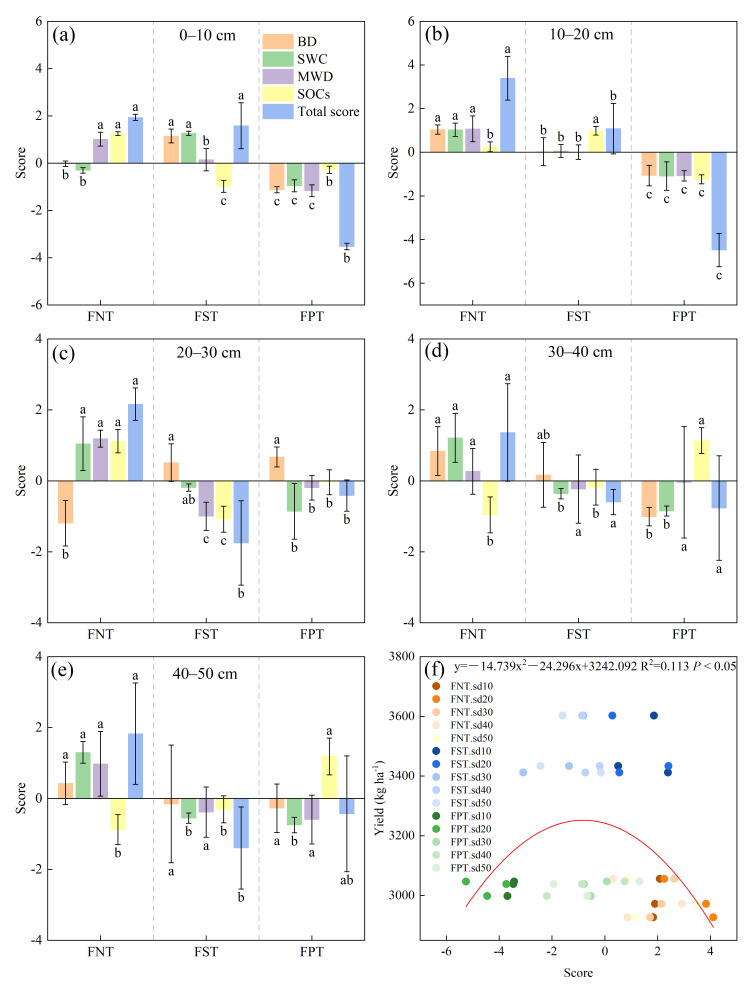
(**a**–**e**) Comprehensive assessment of soil functional quality based on Z-scores at soil depths of 0–50 cm. (**f**) The regression analysis examined the relationship between the Z-scores of five soil layers and yield. Values show the mean (n = 3) ± SD. Significant differences among the treatments on the same indicator are indicated via different lowercase letters (*p* < 0.05; Duncan’s test). sd = soil depth.

As shown in Figure 8a–c,e, Z-scores exhibited considerable variations among tillage treatments (*p* < 0.05), with FNT treatment achieving the highest total Z-scores at soil depths of 0–50 cm. There was no significant difference in the Z-scores between FNT and FST treatments in the 0–10 cm soil layer. FNT primarily relied on significantly increased MWD and SOCs, while FST mainly resulted from significantly reduced BD and increased SWC. FNT significantly increased the Z-scores in the 10–20 cm soil layer primarily through substantial increases in BD, SWC, and MWD, while in the 20–30 cm layer, it primarily relied on significant increases in MWD and SOCs. However, FNT treatment achieved the highest Z-score in the 40–50 cm soil layer primarily by significantly increasing SWC. Regression analysis indicated that the Z-scores exhibited a downward-opening parabolic relationship with yields (Figure 8f). The findings suggested a strong correlation between FST and soil functional quality and yield at soil depths of 30–50 cm, between FNT and soil functional quality and yield at the 0–30 cm soil depths, and between FPT and soil functional quality and yield at the 0–20 cm soil depths. As current data revealed, soil functional quality did not correlate positively with crop yield.

## 3. Discussion

### 3.1. FNT Increased the Content of Large Aggregates, Thereby Enhancing Aggregate Stability

Understanding the long-term effects of various tillage practices on the size distribution and stability of soil aggregates was essential for promoting sustainable crop production [31]. This study indicated that FNT generally significantly increased the content of water-stable aggregates with particle sizes > 2 mm in the 0–30 cm soil depths, compared to FST and FPT. In contrast, FPT markedly increased the content of water-stable aggregates with particle sizes < 0.053 mm in the 0–20 cm soil depths (Figure 2). These results were consistent with the results reported by Ansari et al. [32] and Kan et al. [33], suggesting that FNT contributed to the formation and preservation of larger soil aggregates. The formation of water-stable aggregates primarily relied on chemical forces generated by organic–mineral binding substances. By minimizing long-term soil disturbance, FNT facilitated the continuous accumulation of these binding materials, thereby creating aggregate structures that were more resistant to hydraulic disintegration [34,35]. Furthermore, crop residues retained in the surface layer under FNT not only enhanced microbial activity and promoted soil particle aggregation but also drove macro-aggregate formation by creating pore networks and binding decomposed organic matter [36,37,38]. Soil aggregate stability significantly responded to tillage practices [4]. This study demonstrated that FNT maintained higher water-stable aggregate stability throughout the 0–30 cm soil profile, as reflected by increased MWD and GMD, along with a decreased D (Figure 3). These results aligned with findings from Rieke et al. [39], indicating that reduced tillage significantly enhanced aggregate stability. Zhang et al. [40] also reported that FNT increased MWD by 8.7–42.7% compared to conventional tillage. Two main factors explain these outcomes. Firstly, reduced soil disturbance promoted the formation of larger water-stable aggregates. Secondly, binding agents such as polysaccharides, released by cover crops, actively contributed to aggregate cohesion [41]. Additionally, the input of external organic matter stimulated root growth and secretion of root exudates, which further enhanced aggregate stability [42].

### 3.2. FNT Enhanced Carbon Storage by Protecting AOC Content Within Soil Aggregates

Aggregates of different sizes differentially protected AOC content, collectively regulating the stability and turnover of soil carbon pools [43]. Aggregate stability exhibited a strong correlation with SOC content. Its enhanced stability index was considered a key factor promoting carbon sequestration [44]. This study indicated that FNT and FST generally increased AOC content across all aggregate size fractions (>2 mm, 2–0.25 mm, 0.25–0.053 mm, and <0.053 mm) within the 0–20 cm soil depths, compared to FPT. Conversely, FPT significantly increased AOC content in the >2 mm and 2–0.25 mm aggregate fractions within the 30–50 cm soil depths (Figure 4). These results aligned with the findings of Acharya et al. [45] and Liu et al. [46]. This was due to the role of FNT in reducing SOC mineralization by improving soil structure, controlling erosion, and forming large aggregates [47]. Meanwhile, FPT incorporated straw into deeper soil layers, providing a carbon source for microbial growth and metabolism. This contributed to increased AOC content, particularly large aggregates [48,49]. Our study also indicated that SOCs in the 0–50 cm soil layer under FNT were significantly higher than under FST (8.1%) and FPT (5.8%), due to the increase in SOC content and SOCs at soil depths of 0–30 cm. However, in the 30–50 cm soil depths, FPT significantly increased SOCs by 24.4–30.0% and 14.0–20.0% compared to FNT and FST, respectively (Figure 5b). Feng et al. [50] research supported these results, deep plowing enhanced SOC content in deep soil layers but hindered SOC sequestration across the entire soil profile. However, these findings differed from Alcántara’s research [23], primarily due to variations in deep plowing depth (55–90 cm) and rainfall amounts. Alcántara also emphasized the importance of organic carbon accumulation in the topsoil layer. The amount of soil fixed carbon may depend on specific soil properties, as well as local evapotranspiration rates and rainfall [51]. Although FPT incorporated crop residues into deep soil layers and increased external carbon inputs, simultaneously disrupting soil aggregates [52] and the stable structure of deep soil layers, causing rapid mineralization, thereby leading to SOC content loss and altering carbon distribution within the soil [53].

More importantly, FNT primarily promoted the formation of large aggregates, increasing AOC content within them and thereby enhancing SOC storage [54]. Long-term studies indicated that straw incorporation can enhance carbon sequestration by promoting large aggregate formation and increasing the molecular complexity of organic matter [55]. Exogenous carbon released from straw decomposition entered smaller macro-aggregates before transferring to microaggregates [56]. Notably, although SOC content accumulated in the topsoil under FNT, its exposure to air often accelerated mineralization and decomposition. However, SOC is physically protected within soil aggregates, where it is encapsulated. Consequently, microbial and extracellular enzyme access is reduced, slowing decomposition. Ultimately, SOC content loss is less than the external carbon input. The significant positive correlation between SOC content and AOC content in all particle-sized aggregates across this study further substantiated this point (Figure 6). Additionally, under FNT, SOC content and SOCs were positively correlated with the content of >2 mm aggregates and MWD (Figure 6b,c). Conversely, under FPT, SOC content, and SOCs were positively correlated with content of <0.053 mm aggregates. This also aligned with the pattern that SOC is protected by large aggregates. Meanwhile, the order of Pearson’s correlation coefficients between SOC content and AOC content in four particle size aggregates was FNT > FST > FPT (Figure 6). As can be seen, the high correlation coefficient also confirmed that under FNT, SOC content was primarily regulated by physical protection mechanisms, with its storage being tightly coupled to aggregate structure.

### 3.3. Correlation Between Soil Structure, Crop Yield, and Carbon Sequestration

This study further confirmed that tillage practices significantly affected crop yields by regulating soil physical structure. The FST treatment resulted in the highest grain yield, showing increases of 16.7% and 15.0% compared to FNT and FPT, respectively (Table 1). This was primarily because FST effectively broke the plow pan and increased the proportion of large pores, thereby improving soil structure and enhancing the capacity for deep water storage [57]. These conditions promoted root growth and water utilization, ultimately increasing yield [30]. In contrast, FNT exhibited relatively lower yields. The reason for this phenomenon was that reduced tillage and surface residue cover may lower seedbed temperatures, adversely affecting seedling establishment and panicle formation [27]. Moreover, compacted tillage layers may have restricted root penetration and lateral expansion, thereby impairing water and nutrient uptake. Furthermore, FNT increased carbon sequestration by reducing the biological oxidation of soil organic matter. This may have limited the mineralization of plant nutrients in the soil, resulting in reduced crop yields [51]. Our study showed that the BD was highest under the FNT at a soil depth of 20–30 cm. This effectively explained the negative effects of tillage layer compaction, such as reduced dry matter yield and reduced ear number (Table 1) in winter wheat.

This study found that under FNT and FST, SOC content and SOCs showed positive correlations with the content of >2 mm aggregates, MWD, and the AOC content of all particle sizes (Figure 6b,c). Yield and ear grain number were positively correlated with SP and ear number. SOC content correlated positively with SWC (Figure 6a). These results were in line with previous studies [28,58,59], collectively validating the chain mechanism of “soil structure improvement—optimized water and carbon conditions—yield enhancement”. Generally, increases in SOC content tend to reduce BD. Our study also demonstrated this (Figure 6a). Surprisingly, FNT exhibited high SOC content and BD in the 20–30 cm soil layer. SOC and BD were driven by different factors. The reasons may be that high SOC content likely resulted from the accumulation of previous root residue carbon and the leaching–migration–adsorption precipitationof water-soluble OC; high BD primarily stemmed from mechanical compaction and soil subsidence caused by rainfall. Regarding carbon sequestration, both FNT and FST promoted surface SOC content accumulation by reducing disturbance or incorporating crop residues, though through different pathways. By maintaining surface cover and minimizing soil disturbance, FNT fostered the development and stability of macro-aggregates, thereby improving water retention and carbon sequestration. In contrast, FST incorporated organic matter into deeper soil layers, potentially increasing carbon distribution throughout the soil profile by stimulating deep root growth [60]. Moreover, deep root growth affected water and nutrient uptake, leading to increased yields under FST.

### 3.4. Implications of the Z-Score Method for Agricultural Management Decision-Making

Our study found that the FNT exhibited the highest soil functional quality index in each soil layer (0–50 cm). However, the crop yield under FNT was significantly lower than that of other treatments. This result strongly suggested that soil quality and crop yield did not follow a simple linear relationship (Figure 8f). The potential reason was that FNT not only enhanced aggregate stability within the 0–30 cm soil layers (Figure 3) but also led to increased soil bulk density in the 20–30 cm layer (Figure 1a). This combination of effects increased the difficulty of root penetration through soil layers, thereby leading to reduced water uptake efficiency in deeper roots. The impaired access to deep soil water reserves during the critical grain-filling stage exacerbated late-season drought stress and ultimately limited crop yield. This study excluded soil biological characteristics and lacked consideration of soil’s physical–biological coupling processes, which may also lead to inaccurate assessments of soil functional quality—a limitation of our research. However, FST demonstrated relatively high crop yields and lower soil functional quality scores compared to FNT. FPT showed adverse effects on soil structure and carbon pools [11]. By contrast, FNT demonstrated significant advantages in the comprehensive evaluation, enhancing topsoil SOC content, improving soil structure, and increasing SWC and the proportion of large aggregates. These results underscore the value of FNT in enhancing soil health and strengthening the resilience of agricultural systems. Therefore, agricultural management strategies should shift from focusing on “single-yield maximization” to emphasizing the “optimization of the integrated functions of the soil–crop system”, treating soil as a core asset that requires long-term maintenance. Relying solely on yield as a decision criterion can result in irreversible risks, such as soil degradation. The study found that the FST treatment effectively balanced improvements in deep soil conditions with the maintenance of surface carbon storage and structural stability. This practice enabled the concurrent achievement of both yield increases and soil functional quality enhancement (Figure 8f). In dryland agricultural regions of China, future tillage practices should transition from conventional plowing to conservation tillage systems centered on no-till and subsoiling, thereby promoting sustainable agricultural production.

## 4. Materials and Methods

### 4.1. Experimental Site Description

Our experiment was implemented in Wenxi Experimental Base (110°43′ E, 35°39′ N, altitude 696 m), Yuncheng City, Shanxi Province, which started in 2018. The region is characterized by a typical warm-temperate continental monsoon climate, with mean annual values of 2242 sunshine hours, 12.5 °C temperature, 490 mm precipitation, and a frost-free period averaging 190 days. “Winter wheat–Summer fallow” is the main cultivation system. In July 2018, we recorded the soil type as sandy loam (sand: 57.9%, silt: 18.1%, clay: 18.7%) and the initial physicochemical properties of the topsoil (0–20 cm). Specifically, organic matter, alkali-hydrolysable nitrogen, available phosphorus, available potassium contents, and pH value were 8.8 g kg^−1^, 61.31 mg kg^−1^, 10.4 mg kg^−1^, 114.0 mg kg^−1^, and 8.44, respectively.

### 4.2. Experimental Design

The multi-year fixed-location trial employed a single-factor randomized design with different tillage during the summer fallow, starting in 2018. The annual treatments included the following: (1) FNT—no tillage during the summer fallow; (2) FST—subsoiling tillage during the summer fallow; and (3) FPT—plowing tillage during the summer fallow. Each treatment plot measured 50 m^2^ (5 m × 10 m) and replicated three times. The specific plowing operations are shown in Table 2. After the wheat harvest, the straw was returned to the field. Subsequently, all fields were shallow-rotated once (tillage depth about 10 cm) for moisture conservation. The wheat field was fertilized with 600 kg ha^−1^ of organic fertilizer (N + P_2_O_5_ + K_2_O ≥ 5%, organic matter content ≥ 45%) during the summer fallow period, and humic acid compound fertilizer (N:P_2_O_5_:K_2_O = 21:17:6) was applied according to total 180 kg N (ha^−1^ yr^−1^, 150 kg P ha^−1^ yr^−1^, and 50 kg Kha^−1^ yr^−1^) before sowing. Based on local agricultural production, the winter wheat seeding rate in 2024 was 202 kg ha^−1^. Sowing was performed using a 2BMFD-7/14 full-tillage no-till fertilizer sowing machine (Luoyang Xinle Machinery Equipment Co., Ltd., Luoyang, China). Everything else was consistent with field management.

### 4.3. Sampling and Measurements

After the wheat harvest in June 2025, we collected soil samples at depths ranging from 0 to 50 cm soil depths (10 cm layer interval), and three representative samples were randomly selected and uniformly mixed to form a composite sample. As aggregate samples, we collected undisturbed soil from each treatment plot. The undisturbed soil was broken along natural soil fissures into small fragments < 1 cm in diameter and packed into aluminum lunch boxes. All soil samples were air-dried and cleared of visible gravel and plant debris.

#### 4.3.1. Soil Physicochemical Properties

The soil water content (SWC) was measured by the drying method. Soil bulk density (BD) and soil porosity (SP) were determined via cutting ring approaches. Alkaline nitrogen was determined using the alkaline diffusion method. Available phosphorus was measured using the sodium bicarbonate extraction–molybdenum–antimony colorimetric method. Available potassium was determined via ammonium acetate extraction followed by flame photometry. Soil pH was determined via a pH meter (water/soil = 2.5:1). The SOC content was analysed by dry combustion on a TOC analyzer (Multi N/C 2100, Analytikjena, Jena, Germany). In brief, we soaked 0.5 g of soil in 10 mL 0.5 N HCl for 12 h to ensure carbonate removal prior to measurement [61]. SOC storage (Mg ha^−1^) at 0−50 cm soil depths was calculated by the equivalent soil mass method [62]. SP was calculated using Equation (1):

(1) $$SP=\left(\frac{PD-BD}{PD}\right)\times 100\%$$

where SP is soil porosity (%), and PD is soil particle density (g cm^−3^), for which a reference value of 2.65 g cm^−3^ was used [63].

#### 4.3.2. Determination of Soil Aggregate Characteristics

Next is the determination of soil aggregates by the wet sieve method [64] via the aggregate wet sieve analyzer (LBF-100). In brief, weigh 50 g of soil according to the proportion of different particle sizes after dry sieving, immerse in distilled water for 10 min, shake with an amplitude of 4 cm for 10 min, stew, and then let it sink down for 10 min. The aggregate samples collected from the sieve were placed in aluminium boxes and dried at 50 °C before being weighed. This procedure was repeated three times. The percentage content of water-stable aggregates in the following particle size categories was calculated: >2 mm, 0.25–2 mm, 0.053–0.25 mm, and <0.053 mm. Additionally, the mean weight diameter (MWD), geometric mean diameter (GMD), and fractal dimension (D) of soil aggregates were computed using Equations (2)–(4) [64]. The determination of AOC content at different particle sizes was similar to the determination of SOC content.

(2) $$MWD=\sum_{i=1}^{n}X_{i}\times W_{i}$$

where Xi is the mean diameter of soil aggregates in size fraction i (mm), Wi is the mass proportion of soil particles kept on the sieve for size fraction i, and n is the sum of sieves.

(3) $$GMD=exp(\frac{\sum_{i=1}^{n}W_{i}\log X_{i}}{\sum_{i=1}^{n}W_{i}})$$

where Xi is the mean diameter of each aggregate preserved in each sieve size, Wi refers to the overall dry weight of the aggregate, and n indicates the sum of sieves.

(4) $$\left(3-D\right)\mathrm{lg}\left(\frac{\bar{d_{i}}}{d_{max}}\right)=lg\frac{w(§\leq \bar{d_{i}})}{w_{0}}$$

where $\bar{d_{i}}$ denotes the average diameter (mm) of aggregates in a specific particle size fraction, $w\left(§\leq \bar{d_{i}}\right)$ represents the mass (g) of aggregates with particle sizes $<\bar{d_{i}}$, w_0_ denotes the total mass of aggregates across all particle size classes (g), d_max_ represents the diameter of the largest aggregate (mm), and D is the fractal dimension.

#### 4.3.3. Yield and Its Components

At wheat maturity in 2025, yield components were measured from three randomly selected 1 m^2^ plots per treatment. The measurements included ear number, ear grain number, thousand-grain weight, and grain yield. The yield was expressed at a standard moisture content of 13% [65].

#### 4.3.4. Z-Score

A comprehensive Z-score system was constructed using four independent indicators: physical compactness (BD), water conditions (SWC), aggregate stability (MWD), and carbon storage (SOCs). The Z-score method was employed to standardize the indicator values across five soil layers. BD was treated as a negative indicator; its Z-scores were multiplied by −1 prior to aggregation to ensure that higher values represent better soil conditions. Finally, all standardized scores were summed with equal weighting to generate a total score, with the highest Z-score indicating the most effective tillage management [66]. The formula was as follows:

(5) $$Z_{i}=\left(X_{i}-\bar{X}\right)/SD$$

where Zi is the standardized score, Xi is the measured value of the variable under that treatment, and X is the average value of the variable across all treatments. SD is the standard deviation of the variable across all treatments.

### 4.4. Statistical Analysis

A one-way ANOVA was performed using IBM SPSS Statistics (v.27.0), and significant differences among tillage treatments were identified using Duncan’s tests (*p* < 0.05). Variable importance was assessed for yield and SOCs using random forest modeling in R (v. 4.3.2) software. All figures were generated using Origin (v. 2021) and Adobe Illustrator 2021.

## 5. Conclusions

Compared to FST and FPT, FNT reduced BD at soil depths of 0–20 cm and increased SWC at soil depths of 10–50 cm. It generally enhanced the content of water-stable aggregates with particle size > 2 mm and aggregate stability within the 0–30 cm soil depths, thereby increasing AOC content and ultimately boosting SOC content and storage. However, FST achieved wheat yield increases primarily by enhancing both ear number and ear grain number compared to FNT. The random forest modeling indicated that the most significant factors influencing SOC storage and yield were SOC content and ear number. The Z-score-based assessment of soil functional quality revealed that FNT achieved superiority in enhancing soil structure and carbon sequestration primarily within the 0–30 cm soil layer. In summary, no tillage during the summer fallow was recommended as a superior strategy for enhancing soil sustainability; however, it was crucial to acknowledge the potential for an immediate reduction in crop yield.

## Figures and Tables

**Table 1:** Impact of different summer fallow tillage on winter wheat yield and its component factors in 2025.

Treatment	Ear Number (10^4^ ha^−1^)	Ear Grain Number	Thousand-Grain Weight (g)	Yield (kg ha^−1^)
FNT	558 ± 7.55 c	20.0 ± 0.41 b	25.8 ± 0.42 b	2985 ± 65.43 b
FST	608 ± 4.04 a	22.0 ± 0.22 a	26.8 ± 0.37 ab	3483 ± 104.54 a
FPT	584 ± 6.66 b	20.0 ± 0.35 b	27.5 ± 0.81 a	3028 ± 26.00 b

Note: values show the mean (n = 3) ± SD. Significant differences among the treatments on the same soil layer are indicated via different lowercase letters (*p* < 0.05; Duncan’s test).

**Table 2:** Detailed description of different tillage measures during summer fallow in 2024.

Tillage	Operational Characteristics
No tillage during summer fallow (FNT)	No tillage measures were taken during summer fallow period, time period up to about 102 days, then organic fertilizer was applied on 11 September 2024
Subsoiling tillage during summer fallow (FST)	Operated by a deep pine fertilizer application machine at a depth of about 30–35 cm, while organic fertilizer was applied on 8 August 2024
Plowing tillage during summer fallow (FPT)	Organic fertilizer was applied manually, followed by a deep tiller run to a depth of about 25–30 cm on 8 August 2024

## Data Availability

Data will be made available on request. The data are not publicly available due to the raw data supporting the findings of this study are currently undergoing further analysis.
